# In Vitro Interaction and Killing-Kinetics of Amphotericin B Combined with Anidulafungin or Caspofungin against *Candida auris*

**DOI:** 10.3390/pharmaceutics13091333

**Published:** 2021-08-25

**Authors:** Unai Caballero, Elena Eraso, Guillermo Quindós, Nerea Jauregizar

**Affiliations:** 1 Department of Pharmacology, Faculty of Medicine and Nursing, University of the Basque Country (UPV/EHU), 48940 Leioa, Spain; unai.caballero@ehu.eus; 2 Department of Immunology, Microbiology and Parasitology, Faculty of Medicine and Nursing, University of the Basque Country (UPV/EHU), 48940 Leioa, Spain; elena.eraso@ehu.eus (E.E.); guillermo.quindos@ehu.eus (G.Q.)

**Keywords:** *Candida auris*, combination, antifungal agents, amphotericin B, echinocandins, anidulafungin, caspofungin, time-kill

## Abstract

Treatment of invasive infections caused by *Candida auris* is challenging due to the limited therapeutic options. The combination of antifungal drugs may be an interesting and feasible approach to be investigated. The aim of this study was to examine the in vitro activity of amphotericin B in combination with anidulafungin or caspofungin against *C. auris*. In vitro static time–kill curve experiments were conducted for 48 h with different combinations of amphotericin B with anidulafungin or caspofungin against six blood isolates of *C. auris*. The antifungal activity of 0.5 mg/L of amphotericin B was limited against the six isolates of *C. auris*. Similarly, echinocandins alone had a negligible effect, even at the highest tested concentrations. By contrast, 1 mg/L of amphotericin B showed fungistatic activity. Synergy was rapidly achieved (8 h) with 0.5 mg/L of amphotericin B plus 2 mg/L of anidulafungin or caspofungin. These combinations lead to a sustained fungistatic effect, and the fungicidal endpoint was reached against some *C. auris* isolates. Additionally, ≥0.5 mg/L of either of the two echinocandins with 1 mg/L of amphotericin B resulted in fungicidal effect against all *C. auris* isolates. In conclusion, combinations of amphotericin B with anidulafungin or caspofungin provided greater killing with a lower dose requirement for amphotericin B compared to monotherapy, with synergistic and/or fungicidal outcomes.

## 1. Introduction

*Candida auris* is a multi-drug resistant fungal pathogen, responsible for several cases of invasive fungemia since 2009, that has become a global public concern [1,2]. In contrast to what has been observed for other species of *Candida*, where resistance to antifungal drugs are exceptions, in the case of *C. auris*, both intrinsic and acquired resistance are the norm [3,4]. *C. auris* shows high rates of resistance to fluconazole and other azoles, along with a reduced susceptibility to amphotericin B and echinocandins that complicate the therapeutic approaches for candidiasis caused by this species [5,6]. Additionally, cross- and multi-resistance has been found in 18–41% of *C. auris* isolates and approximately 4% of them are resistant to the three main classes antifungal drugs: polyenes, azoles, and echinocandins [7,8]. Even echinocandins (anidulafungin, caspofungin, and micafungin) are nowadays considered the first-line therapeutic option to treat *C. auris* infections, therapeutic failures often occur in critically ill patients. It has been evidenced that echinocandins are not fungicidal against *C. auris* clinical isolates representing the four identified main clades [9,10]. Against a background of increasing evidence of limited susceptibility of *C. auris* to available antifungal agents in monotherapy, the approach of combining drugs with different antifungal mechanisms has been proposed to optimize the therapeutic management of invasive mycoses.

Echinocandins act as non-competitive inhibitors of β-(1,3)-D-glucan synthase, involved in the biosynthesis of β-(1,3)-D-glucan in the fungal cell wall. Amphotericin B binds to ergosterol, forming pores and disrupting fungal cell membrane. It has been demonstrated that amphotericin B exhibits in vitro fungicidal activity against *C. auris*, albeit at high concentrations [9]. Recent studies included combinations of azoles, echinocandins, amphotericin B, and flucytosine, with disparate results of indifference or synergy, depending on the combined drugs and tested isolates of *C. auris* [11,12,13,14]. In this line, the in vitro combinations of echinocandins and isavuconazole against *C. auris* have shown synergism [15,16]. Hence, the combinations of the first-line echinoncadins with the fungicidal agent amphotericin B against *C. auris* deserves to be investigated.

The aim of the current study was to examine the in vitro activity of the combination of amphotericin B with anidulafungin or caspofungin, against *C. auris*. To our knowledge, this is the first study that has investigated the combination of amphotericin B with echinocandins against this pathogen using time-kill curves.

## 2. Materials and Methods

Six blood isolates of *C. auris* (CJ94, CJ97, CJ98, CJ99, CJ100, and CJ102) collected from different patients from an invasive candidiasis outbreak in the Hospital Universitario y Politécnico La Fé (Valencia, Spain) were studied [17]. The isolates of the study were phylogenetically close to the South African clade isolates [17,18]. The *C. auris* blood isolates used in this study were non-aggregating isolates, as it was previously demonstrated in vitro [19]. Fungal strains were stored in vials with sterile distilled water and cultivated in Sabouraud dextrose agar (SDA), as previously described [14]. MICs were determined following EUCAST E.DEF 7.3 document [20]. MICs for amphotericin B, anidulafungin, and caspofungin were 1, 0.125, and 0.25 mg/L, respectively, for all isolates.

Amphotericin B (Sigma-Aldrich, Madrid, Spain), anidulafungin (Pfizer SLU, Madrid, Spain), and caspofungin (Merck Sharp and Dohme, Madrid, Spain) were obtained in powder, dissolved in dimethyl sulfoxide (DMSO) to obtain stock solutions of 3200 mg/L and maintained at −80 °C until use.

Time–kill assays were carried out as previously described in a 96-well flat-bottomed microtiter plates, in RPMI medium, and by using an inoculum size of 1–5 × 10^5^ colony forming units (CFU/mL) [15]. Antifungal drug concentrations assayed were selected on the basis of previous time–kill and checkerboard results (unpublished data). Accordingly, 0.5 mg/L of amphotericin B was combined with 0.5, 1, and 2 mg/L of each echinocandin, and 1 mg/L of amphotericin B was combined with 0.25, 0.5, and 1 mg/L of echinocandin. Aliquots were collected at 0, 2, 4, 6, 8, 24, and 48 h for colony counts, plated onto SDA in triplicate and incubated at 37 °C for 24 to 48 h. Depending on the drug concentration and expected activity, samples were either first diluted in PBS (no antifungal activity) or plated directly (5–20 µL). When a sterilizing activity was expected, the whole well (200 µL) was sampled onto a SDA plate. Therefore, the lower limit of detectable colony counts was 5 CFU/mL. The carryover effect was determined as previously described [21]. All experiments were conducted twice. Fungistatic or fungicidal activities were defined as a <3 log or ≥3 log reduction in CFU/mL, respectively. Synergism was defined as a difference of >2 log between the activity of the drugs in combination and the activity of the most active agent in monotherapy [22].

Time–kill curves were analysed as described previously [15], by fitting the CFU/mL observations to an exponential equation: N_t_ = N_0_ × e^kt^ (N_t_, number of CFU/mL at time t; N_0_, starting inoculum; k, growing or killing rate constant, and t, incubation time). This equation was linearized by applying natural logarithms, and k values were then used to compare the killing activities among drugs and concentrations. Positive killing rate (k) values indicate growth, and negative values show killing. Thus, the seven time-points on each killing curve were reduced to one k value (mean values). Goodness of fit for each combination was assessed by the r^2^ value (≥0.8). Significant differences in killing kinetics among combinations and concentrations were evaluated by ANOVA with Bonferroni’s post-testing (GraphPad Prism 5.01). A *p* value < 0.05 was considered significant.

## 3. Results

**Figure 1:**
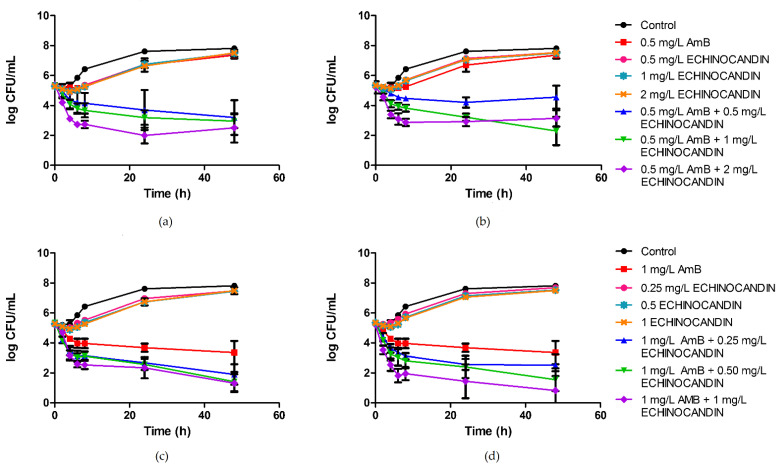
Time–kill curves showing the effects over 48 h of amphotericin B and echinocandins, in monotherapy and in combination, against *Candida auris*: (**a**) 0.5 mg/L of amphotericin B and anidulafungin; (**b**) 0.5 mg/L of amphotericin B and caspofungin; (**c**) 1 mg/L of amphotericin B and anidulafungin; (**d**) 1 mg/L of amphotericin B and caspofungin. Data are plotted as average data points from the different isolates ± standard error (SE). Antifungal drug concentrations are expressed in mg/L. AmB, amphotericin B.

Mean time–kill curves for all isolates and combinations are shown in Figure 1. No antifungal carryover was observed. Fungal counts at 8, 24, and 48 h for each isolate and combination are depicted in Table 1. The antifungal activity of amphotericin B alone at the concentration of 0.5 mg/L was limited against the six isolates of *C. auris*, as no fungicidal nor fungistatic effect was achieved. Similarly, echinocandins alone had a negligible effect, even at the highest tested concentrations. In contrast to the poor results of the monotherapies, the combination of 0.5 mg/L of amphotericin B with anidulafungin or caspofungin led to a sustained fungistatic effect, and the fungicidal endpoint was reached against isolates *C. auris* CJ94, *C. auris* CJ99, *C. auris* CJ100, and *C. auris* CJ102. The interactions were synergistic from 24 h onwards for all isolates and concentrations. Conversely, 1 mg/L of amphotericin B showed fungistatic activity, and the combinations of this polyene with the echinocandins were mostly additive. Regardless of the additivity detected, when ≥0.5 mg/L of echinocandin was combined with 1 mg/L of amphotericin B, fungicidal effect was achieved against all isolates.

Interestingly, a quarter of all combination time–kill experiments showed regrowth phenomena. The regrowth was observed specially for the combinations of 0.5 mg/L of amphotericin B with 2 mg/L of echinocandin, which resulted, in the case of caspofungin, in a lower mean effect compared to the combination that included 1 mg/L of caspofungin (Figure 1).

**Figure 2:**
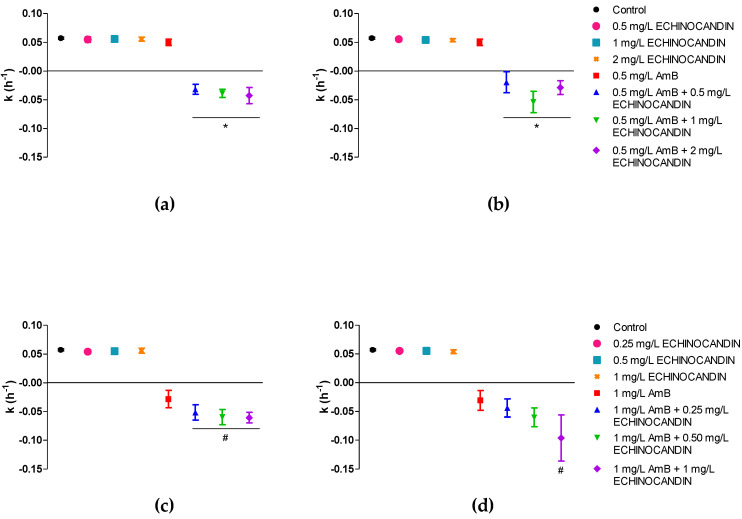
Mean killing-rate constant values for the combinations of amphotericin B and echinocandins against *C. auris*: (**a**) 0.5 mg/L of amphotericin B and anidulafungin; (**b**) 0.5 mg/L of amphotericin B and caspofungin; (**c**) 1 mg/L of amphotericin B and anidulafungin; (**d**) 1 mg/L of amphotericin B and caspofungin Each data point represents the mean result ± standard deviation (error bars) of all isolates and replicates. * *p* < 0.05 vs. 0.5 mg/L AmB; # *p* < 0.05 vs. 1 mg/L AmB (One-way ANOVA followed by Bonferroni’s post hoc test). AmB, amphotericin B.

When the killing-rate constants were analysed, positive k values, non-different from control curves, were obtained for all the drugs and concentrations in monotherapy (k = 0.055 h^−1^), except for amphotericin B at 1 mg/L, with a negative mean k value (k = −0.031 h^−1^), indicating fungal killing, even though the fungicidal threshold was not reached (Table 1). In contrast to the positive k values for echinocandins alone, and for 0.5 mg/L of amphotericin B, the time–kill curves patterns shifted for the combinations, all killing-rate constants were negative and significantly different from monotherapy. This was also the case for the combinations of anidulafungin with 1 mg/L of amphotericin B. Conversely, only the k of the combination of 1 mg/L amphotericin B plus 1 mg/L of caspofungin was significantly different from amphotericin B monotherapy. The reason may be due to the higher variability observed in the activity of caspofungin in contrast to anidulafungin. Mean killing rate constants for each drug combination are graphically represented in Figure 2.

## 4. Discussion

Although echinocandins are the first-choice treatment for invasive candidiasis, the present study found that anidulafungin and caspofungin in monotherapy had no activity against any of the six *C. auris* isolates studied, while amphotericin B did, although at concentrations ≥1 μg/mL. Previously, we have reported that amphotericin B reached fungicidal endpoint at concentrations ≥2 mg/L [23], in concordance with another study [9]. Thus far, there are only two works that have evaluated the activity of echinocandins against *C. auris* with this time–kill curve approach. In both studies, the first in vitro evidence of the lack of fungicidal effect of the echinocandins was provided [9,10]. Dudiuk et al. reported that the average k values for caspofungin and anidulafungin were close to zero. Our results are similar, especially for caspofungin at 48 h, although the activity of anidulafungin in their study was higher, as they reported a fungistatic effect at 24 and 48 h [9]. Kovács et al. investigated the killing activities of the three echinocandins against isolates from each *C. auris* clade. Their results were mostly in agreement with Dudiuk et al. and with those reported in the current study. They found fungistatic activity against the isolates from all clades [10]. Different possible explanations for the weak in vitro fungistatic activity of echinocandins against *C. auris* have been proposed, such as the cell aggregation formation as a survival strategy [10]. Szekely et al. observed the in vitro formation of large cell aggregates in South African isolates [24], and other reports have also described this *C. auris* aggregative behaviour in vivo for most (up to 84%) isolates from the South African clade [25,26]. Interestingly, this aggregation was induced and reversible by echinocandin exposure, but not with amphotericin B exposure [24]. However, we have previously reported that isolates from our study related to the South African clade do not grow as aggregates and are more pathogenic in vivo than other isolates that displayed an aggregating phenotype [19]. This could be due to the fact that *C. auris* produces virulence factor in a strain-dependent way, with different aggregating behaviour and virulence among isolates within the same clade [25,26]. In fact, differences in murine virulence have been reported for isolates from the same clade [26]. Nevertheless, regarding the clinical relevance of the aggregating ability of certain *C. auris* isolates, further studies are needed to determine if the aggregate formation during infection protects those strains from the effect of antifungal drugs.

Additionally, it has been suggested that the aggregation may not be enough for *C. auris* isolates to overcome echinocandin exposure [10], while other mechanisms have also been proposed to explain the limited fungistatic activity of echinocandins, such as the increased cell wall chitin as a response to the decrease in the amount of β-glucan induced by the echinocandin exposure [27,28]. In this line, other reports found increased chitin amounts in *C. auris*, compared to other species more susceptible to echinocandin drugs [29].

The lack of fungicidal activity found in vitro for echinocandins against *C. auris*, along with the high concentrations of amphotericin B required to reach the fungicidal endpoint, support the interest to examine the combinations with the two classes of antifungal agents.

In the current study it should be highlighted that synergy was rapidly achieved (8 h) with the combinations of amphotericin B at 0.5 mg/L and the highest concentration of anidulafungin or caspofungin (2 mg/L). Once achieved, synergy was sustained over 48 h. Moreover, the combinations of amphotericin B at 1 mg/L and anidulafungin or caspofungin resulted in a fungicidal activity. The achievement of this fungicidal activity and the earlier arrival to this point was related to higher concentrations of echinocandins. The findings of synergy, and in some cases of fungicidal activity, were promising, especially if the lack of activity of the drugs alone (except for amphotericin B at the high concentration) is considered.

To our knowledge, this is the first study on the combination of amphotericin B with echinocandins against *C. auris* through in vitro time–kill curves. Very few studies have examined the effect of antifungal drug combinations against this species, and most of them have assessed the interactions solely with checkerboard data and fractional inhibitory concentration index (FICI) determination. O’Brien et al. studied *C. auris* isolates from a New York outbreak of candidiasis and found synergism for the combination of flucytosine and echinocandins or amphotericin B, but not for the combination of amphotericin B with echinocandins [13]. The New York strains that they evaluated were related to the South Asian clade, while our isolates from the Valencia outbreak are related to the South African clade. This different origin could explain the lack of concordance between both studies. However, Bidaut et al. observed indifference for the combination of amphotericin B with flucytosine tested against *C. auris* isolates determined by FICI [12]. Published works suggest that there might be differences in antifungal susceptibility among *C. auris* clades [10,12]. Therefore, the synergism and antifungal activity shown by the combinations of amphotericin B and anidulafungin or caspofungin in our study may not replicate in isolates belonging to other clades.

Combination therapy with amphotericin B and echinocandins has been studied for other species of *Candida*, with variable results. Kiraz et al. reported that amphotericin B plus caspofungin showed synergism against 46% of the tested *Candida glabrata* isolates with time–-kill assays [30]. Another study found a similar degree of synergism with anidulafungin against various species of *Candida* [31]. Conversely, other studies with *C. glabrata* demonstrated a lack of synergy with the combinations of amphotericin B and echinocandins [32]. Although most interactions were deemed indifferent by the checkerboard method in the study of Serena et al. on the combination of amphotericin B with micafungin, there was synergism against some strains that were further analysed by time–kill methodology and the combination demonstrated a fast-killing activity against them [33].

Olson et al. tested the combinations of amphotericin B and caspofungin or micafungin in immunosuppressed mice infected with *C. glabrata* and found that both combinations, administered either concomitantly or sequentially, significantly reduced the fungal burden in tissues compared to any of the drugs in monotherapy [34]. Hossain et al. also reported a significant fungal reduction with the co-administration of amphotericin B and caspofungin against azole-resistant *Candida albicans*. However, when mice survival was checked, the difference between combination therapy and monotherapy with amphotericin B was not significant, even though the survival was higher in the drug combination group [35].

Clinical evidence regarding the combination of amphotericin B with echinocandins for invasive candidiasis is scarce and mostly published as case reports. Mpakosi et al. reported the successful treatment with liposomal amphotericin B and micafungin of a preterm infant with *Candida (Metschnikowia) pulcherrima* fungaemia [36]. This rare species is, along with *C. auris*, part of the *Metschnikowiaceae* family. Another report from Japan described the eradication of *Candida guillermondii* infection in an oncology patient using the same combination [37]. Roberts et al. reported a case of a patient suffering from *C. auris* intra-articular infection successfully treated with amphotericin-impregnated spacer in addition to systemic fluconazole or micafungin [38]. Apart from these case reports, there is limited evidence on this issue, and the combined therapy of echinocandins with amphotericin B has been used mostly in the treatment of invasive aspergillosis. Yilmaz et al. observed that the combination of amphotericin B and caspofungin was safe and effective in the treatment of invasive fungal infections of children with haematological malignancy, refractory to amphotericin B, and caused by *Aspergillus* or *Candida* [39]. The multicentre observational ProCAS study evaluated the effectiveness and safety of caspofungin in monotherapy and in combination with amphotericin B or voriconazole in adult haematological patients with invasive candidiasis. Favourable results were reported for *Candida krusei* fungemia treated with amphotericin B plus caspofungin [40].

## 5. Conclusions

The combinations of amphotericin B with echinocandins provided greater killing with a lower dose requirement of amphotericin B against all isolates of *C. auris*. These findings could support a new therapeutic approach and expand the therapeutic options by combining two first-line antifungal drugs in those cases where invasive candidiasis caused by this species does not respond to current treatment. Despite the favourable results of the combination, further in vivo studies are needed to better assess possible clinical relevance, considering criteria of effectiveness and safety.

## Figures and Tables

**Table 1:** Fungal counts at 8, 24, and 48 h and interaction classification.

	AmB+ECH (mg/L)	Fungal Count (log CFU/mL) (SD)					
		AmB+ANF			AmB+CSP		
		8 h	24 h	48 h	8 h	24 h	48 h
CJ94	Control	6.68 (0.26)	7.65 (0.08)	7.83 (0.10)	6.68 (0.26)	7.65 (0.10)	7.83 (0.10)
	0.5 AMB	5.14 (0.29)	6.58 (0.43)	7.37 (0.14)	5.14 (0.29)	6.58 (0.43)	7.37 (0.14)
	0.5+0.5	4.52 (0.26)	**3.91 (0.05)**	**3.62 (0.58)**	4.62 (0.07)	**4.36 (0.40)**	**5.26 (0.10)**
	0.5+1	3.73 (0.31)	**3.72 (0.31)**	**2.86 (0.66)**	4.10 (0.00)	**3.22 (0.25)**	**3.96 (0.51)**
	0.5+2	**2.92 (0.16)**	**1.10 (1.55)**	**1.07 (1.51)**	**2.98 (0.30)**	**3.21 (0.45)**	**3.20 (0.08)**
	1 AMB	4.03 (0.23)	3.40 (0.21)	2.52 (0.06)	4.03 (0.23)	3.40 (0.20)	2.52 (0.06)
	1+0.25	3.15 (0.49)	2.90 (0.71)	1.35 (0.21)	3.12 (0.17)	2.37 (0.11)	2.68 (0.19)
	1+0.5	3.13 (0.18)	2.21 (0.17)	0.84 (1.19)	3.07 (0.10)	2.60 (0.69)	1.71 (1.39)
	1+1	2.87 (0.03)	2.66 (0.04)	1.71 (0.56)	2.50 (0.14)	2.09 (0.84)	2.62 (0.12)
CJ97	Control	6.20 (0.00)	7.62 (0.06)	7.71 (0.3)	6.20 (0.00)	7.63 (0.06)	7.71 (0.30)
	0.5 AMB	5.23 (0.05)	6.81 (0.04)	7.32 (0.00)	5.23 (0.05)	6.81 (0.04)	7.32 (0.00)
	0.5+0.5	4.67 (0.00)	**4.21 (0.02)**	**3.54 (0.18)**	4.65 (0.02)	**4.29 (0.66)**	**4.08 (0.36)**
	0.5+1	3.65 (0.07)	**3.32 (0.04)**	**2.77 (0.54)**	3.98 (0.02)	**3.47 (0.16)**	**2.52 (0.11)**
	0.5+2	**3.08 (0.39)**	**2.49 (0.86)**	**2.44 (2.39)**	3.31 (0.25)	**3.20 (0.29)**	**3.40 (0.22)**
	1 AMB	4.42 (0.32)	4.16 (1.03)	3.20 (0.69)	4.43 (0.32)	4.17 (1.00)	3.20 (0.69)
	1+0.25	3.68 (0.25)	2.72 (0.22)	2.63(1.99)	3.52 (0.17)	3.07 (0.04)	2.48 (0.70)
	1+0.5	3.30 (0.00)	3.28 (0.47)	**0.50 (0.70)**	3.45 (0.21)	2.44 (0.22)	1.76 (1.00)
	1+1	2.83 (0.14)	2.87 (1.37)	2.09 (0.84)	**2.17 (0.17)**	2.43 (0.21)	2.37 (3.35)
CJ98	Control	6.38 (0.02)	7.54 (0.01)	7.95 (0.09)	6.38 (0.02)	7.54 (0.01)	7.95 (0.08)
	0.5 AMB	5.20 (0.05)	6.40 (0.40)	7.50 (0.16)	5.23 (0.05)	6.4 (0.43)	7.48 (0.16)
	0.5+0.5	4.42 (0.20)	**3.79 (0.39)**	**3.55 (0.14)**	4.38 (0.16)	**3.96 (0.22)**	**5.08 (1.17)**
	0.5+1	3.35 (0.02)	**3.03 (0.37)**	**3.00 (1.00)**	3.55 (0.44)	**3.22 (0.49)**	**1.85 (0.21)**
	0.5+2	**2.86 (0.05)**	**3.23 (0.78)**	**3.39 (1.12)**	**2.73 (0.05)**	**2.48 (0.05)**	**3.52 (1.03)**
	1 AMB	3.85 (0.07)	3.61 (0.48)	3.77 (0.46)	3.85 (0.07)	3.61 (0.48)	3.77 (0.46)
	1+0.25	3.13 (0.28)	2.55 (0.40)	1.79 (0.49)	2.95 (0.12)	2.57 (0.13)	3.60 (0.73)
	1+0.5	3.06 (0.09)	2.39 (0.80)	1.79 (1.40)	3.00 (0.01)	3.31 (0.59)	**1.00 (0.01)**
	1+1	2.26 (0.15)	3.22 (2.00)	**0.60 (0.84)**	**1.3 (0.42)**	1.97 (2.78)	**0.00 (0.00)**
CJ99	Control	6.88 (0.44)	7.54 (0.04)	7.93 (0.09)	6.88 (0.44)	7.54 (0.04)	7.93 (0.09)
	0.5 AMB	5.30 (0.37)	7.05 (0.10)	7.54 (0.01)	5.30 (0.37)	7.05 (0.11)	7.54 (0.01)
	0.5+0.5	4.68 (0.20)	**4.68 (0.60)**	**3.28 (1.16)**	4.70 (0.14)	**3.94 (0.17)**	**3.19 (0.84)**
	0.5+1	3.88 (0.16)	**2.68 (0.27)**	**3.11 (0.87)**	3.75 (0.11)	**2.84 (0.42)**	**1.68 (0.01)**
	0.5+2	**2.58 (0.16)**	**1.7 (0.49)**	**2.40 (0.80)**	**2.66 (0.23)**	**2.69 (1.68)**	**3.40 (2.35)**
	1 AMB	3.88 (0.26)	2.99 (0.43)	4.93 (0.42)	3.88 (0.25)	2.99 (0.43)	4.94 (0.41)
	1+0.25	2.90 (0.59)	2.23 (1.12)	**1.77 (1.96)**	3.10 (0.47)	1.99 (1.33)	**1.43 (0.98)**
	1+0.5	2.90 (0.47)	2.29 (1.76)	**1.91 (1.65)**	2.38 (0.54)	2.06 (0.06)	**1.90 (1.63)**
	1+1	2.25 (0.35)	2.23 (0.24)	**0.95 (1.34)**	2.25 (0.49)	2.19 (0.15)	**0.00 (0.00)**
CJ100	Control	6.43 (0.33)	7.7 (0.19)	7.81 (012)	6.43 (0.33)	7.70 (0.20)	7.80 (0.12)
	0.5 AMB	5.50 (0.47)	7.28 (0.06)	7.49 (0.04)	5.50 (0.47)	7.28 (0.06)	7.49 (0.04)
	0.5+0.5	4.55 (0.35)	**4.72 (0.2)**	**4.32 (1.43)**	4.55 (0.26)	**4.78 (0.03)**	**3.26 (0.14)**
	0.5+1	3.02 (0.96)	**2.65 (0.91)**	**2.29 (0.40)**	3.82 (0.68)	**2.75 (0.43)**	**1.42 (0.98)**
	0.5+2	**2.66 (0.53)**	**2.25 (0.23)**	**1.74 (0.55)**	**2.70 (0.00)**	**2.93 (0.08)**	**2.09 (0.74)**
	1 AMB	4.17 (0.19)	3.34 (0.49)	3.10 (0.00)	4.16 (0.18)	3.34 (0.49)	3.10 (0.00)
	1+0.25	2.96 (1.36)	2.38 (1.33)	**1.07 (1.52)**	3.02 (0.74)	2.67 (0.49)	2.10 (0.18)
	1+0.5	3.20 (0.98)	2.45 (0.51)	2.1 (0.72)	2.88 (0.82)	**1.23 (1.74)**	**0.53 (0.74)**
	1+1	2.61 (0.11)	1.37 (1.59)	1.73 (1.41)	**1.80 (0.00)**	**0.00 (0.00)**	**0.00 (0.00)**
CJ102	Control	6.52 (0.16)	7.59 (0.15)	7.59 (0.23)	6.52 (0.16)	7.59 (0.15)	7.6 0(0.24)
	0.5 AMB	5.11 (0.21)	6.06 (0.12)	6.94 (0.57)	5.11 (0.21)	6.06 (0.13)	6.94 (0.57)
	0.5+0.5	4.46 (0.18)	**4.04 (0.40)**	**4.01 (0.15)**	4.25 (0.16)	**3.86 (0.16)**	**4.58 (0.48)**
	0.5+1	4.40 (0.14)	**3.73 (0.09)**	**3.69 (0.43)**	3.91 (0.02)	**3.36 (0.01)**	**2.39 (0.00)**
	0.5+2	**2.65 (0.35)**	**2.00 (0.25)**	**3.52 (0.00)**	**2.70 (0.14)**	**2.62 (0.52)**	**3.06 (0.00)**
	1 AMB	3.80 (0.14)	3.82 (0.44)	4.04 (0.19)	3.80 (0.14)	3.82 (0.43)	4.04 (0.18)
	1+0.25	3.05 (0.44)	2.73 (0.36)	2.79 (1.14)	2.94 (0.27)	3.56 (0.51)	3.31 (0.00)
	1+0.5	3.22 (0.40)	2.52 (0.84)	**1.39 (0.52)**	2.21 (0.50)	2.92 (1.10)	2.88 (0.10)
	1+1	2.39 (0.06)	**1.74 (1.02)**	**0.84 (1.18)**	**1.70 (0.70)**	**0.00 (0.00)**	**0.00 (0.00)**

Synergy is marked in bold and fungicidal effects are underlined. AmB: amphotericin B; ECH: echinocandin; ANF: anidulafungin; CSP: caspofungin.
